# Time Domain Transmissiometry-Based Sensor for Simultaneously Measuring Soil Water Content, Electrical Conductivity, Temperature, and Matric Potential

**DOI:** 10.3390/s23042340

**Published:** 2023-02-20

**Authors:** Yuki Kojima, Manabu Matsuoka, Tomohide Ariki, Tetsuo Yoshioka

**Affiliations:** 1 Department of Civil Engineering, Gifu University, 1-1 Yanagido, Gifu-shi 501-1193, Gifu, Japan; 2 Sensing Systems Engineering Div. 2, DENSO CORPORATION, 1-1 Showa-cho, Kariya-shi 448-8661, Aichi, Japan

**Keywords:** time domain transmissiometry, soil water content, soil bulk electrical conductivity, soil temperature, soil matric potential

## Abstract

Owing to the increasing popularity of smart agriculture in recent years, it is necessary to develop a single sensor that can measure several soil properties, particularly the soil water content and matric potential. Therefore, in this study, we developed a sensor that can simultaneously measure soil water content (θ), electrical conductivity (σ_b_), temperature, and matric potential (ψ). The proposed sensor can determine θ and σ_b_ using time domain transmissiometry and can determine ψ based on the capacitance of the accompanying ceramic plate. A series of laboratory and field tests were conducted to evaluate the performance of the sensor. The sensor output values were correlated with the soil properties, and the temperature dependence of the sensor outputs was evaluated. Additionally, field tests were conducted to measure transient soil conditions over a long period. The results show that the developed sensor can measure each soil property with acceptable accuracy. Moreover, the root-mean-square errors of the sensor and reference values were 1.7 for the dielectric constant (which is equivalent to θ), 62 mS m^−1^ for σ_b_, and 0.05–0.88 for log ψ. The temperature dependence was not a problem, except when ψ was below −100 kPa. The sensor can be used for long-term measurements in agricultural fields and exhibited sufficient lifetime and performance. We believe that the developed sensor can contribute to smart agriculture and research on heat and mass transfer in soil.

## 1. Introduction

Smart farming, which uses sensing devices as well as information and communication technologies to precisely manage field conditions, has gained widespread attention in recent years [1]. Researchers have developed several types of sensors owing to their increasing use in smart farming, e.g., sensors for capturing farming fields and crop conditions. Among the currently available sensors, some measure soil information, including the soil temperature, volumetric water content (θ), bulk electrical conductivity (σ_b_), and matric potential (ψ).

Soil temperature partially controls seed germination and plant growth [2], and can be easily measured using thermistors and thermocouples. The θ value is often considered to be the most important parameter for smart farming, as it determines the amount of water that exists in the soil and supports decision-making regarding the amount and timing of irrigation [3,4,5]. Therefore, many soil moisture sensors have been developed and are commercially available. For example, time domain reflectometry (TDR), which measures the soil bulk dielectric constant (ε_b_) from the travel time of electromagnetic pulses through the guide rods and converts ε_b_ to θ, is frequently used in research projects [6,7,8]. Relatively low-cost soil moisture sensors often provide data based on measurements of the soil capacitance, which is strongly correlated with θ [9,10,11]. The conductivity σ_b_ is measured when the amount and timing of fertilizer application is precisely controlled, or when the soil salinity needs to be monitored [12,13,14]. The σ_b_ value is measured from the attenuation of the electromagnetic pulse using the TDR system [15,16] or the four-electrode method [17,18], which is sometimes used in conjunction with capacitance sensors [10]. The parameter ψ is the energy state of water within the soil pores associated with capillary and adsorptive forces between soil particles and water [19], and while it is not often focused upon, it is another important property for determining water availability for plants. Since ψ determines whether plants can take up water from the soil, its measurement must be included in smart farming systems [20]. The ψ value is measured using a tensiometer, which is a conventional technique for measuring water pressure equilibrated with soil pore water via a porous cup, or porous medium-based sensors [21,22]. The porous medium-based sensors, which measure the electrical or thermal properties of porous media inserted into the soil and equilibrated soil pore water, are becoming the most common tools for obtaining ψ [20,23,24,25].

In smart farming, various soil properties are measured at several depths and locations, and this requires many sensors. As mentioned previously, several sensors have been developed for each soil property. However, using a large number of sensors can make the system setup laborious, thereby increasing the cost and vulnerability of the system. Therefore, sensors that measure multiple physical properties with a single probe are desirable. Some sensors have certain abilities; for example, TDR can measure θ and σ_b_ [15,16]. Some other capacitance sensors can be combined with the four-electrode method and thermistors [10]. However, only a few sensors simultaneously measure the two most important soil properties, θ and ψ. Some researchers have used TDR sensors combined with a gypsum block or tensiometer [26,27] to measure these two properties. However, these sensors reduce the ability of the TDR to measure σ_b_. Kojima et al. [28] combined a heat pulse probe [29,30] and a porous medium to measure the soil thermal properties, θ, and ψ. However, the sensor’s performance under field conditions is unknown. Therefore, it is necessary to develop a sensor that can simultaneously measure multiple soil properties, including θ and ψ. 

In this study, we developed a new sensor that simultaneously measures θ, σ_b_, soil temperature, and ψ, and evaluated its performance. Furthermore, we implemented time domain transmissiometry (TDT) to measure θ and σ_b_ using the proposed sensor. TDT is a technique similar to TDR, wherein an electromagnetic pulse propagates the “looped” guide rods, and its travel time and attenuation are converted into θ and σ_b_ [31,32,33]. TDT has certain advantages over TDR. For example, the time-domain waveform for travel time determination is easier to analyze than TDR. Therefore, automated water content measurement is more likely to be stable and accurate [34]. Additionally, TDT systems can reportedly be implemented at a low cost [35]. In this study, the TDT technique was combined with a capacitance sensor with a ceramic plate to measure ψ, and a thermistor was provided for the proposed sensor.

## 2. Materials and Methods

### 2.1. Sensor Development

**Figure 1:**
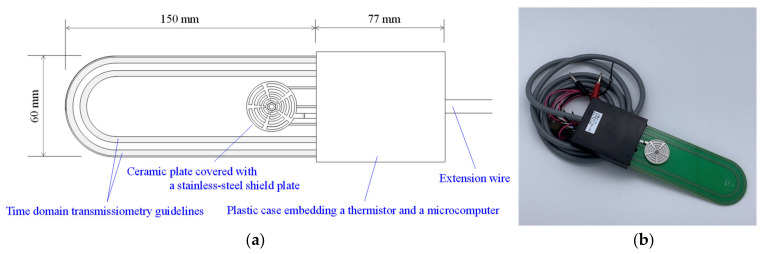
(**a**) Schematic and (**b**) photograph of the proposed sensor.

Figure 1 shows a schematic and photograph of the proposed sensor manufactured by DENSO. The sensor comprises a 150 mm long, 60 mm wide, and 1.6 mm thick printed circuit board; a 77 mm long, 66 mm wide, and 19 mm thick plastic case; and an extension wire. The looped guidelines for TDT electromagnetic pulse propagation were printed on the circuit board, and a ceramic plate was embedded to measure ψ. A capacitor was printed behind the ceramic plate, which was covered with a stainless-steel shield plate. The stainless-steel shield comprised many holes to allow the ceramic plate to contact the soil, and it was grounded. The looped guide and ceramic plate were embedded on both sides of the substrate to obtain an average of the soil properties at these two points. A thermistor, a microcomputer, and integrated circuits for the TDT and capacitance measurements were embedded in a plastic case filled with polyurethane resin. The temperature measured with the thermistor is affected by the thermal conductance of the plastic box as there may be a slight delay in the temperature response of the sensor. The extension wire can be connected to a personal computer or a wireless communication module.

**Figure 2:**
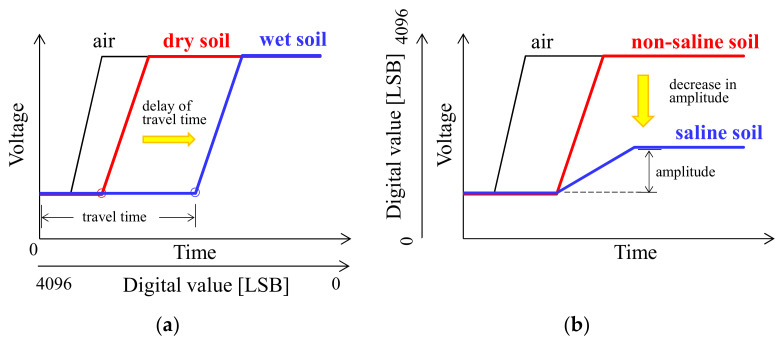
Time domain transmissiometry waveform analysis for (**a**) soil water content (dielectric constant) and (**b**) electrical conductivity.

The microcomputer and integrated circuits control the transmission and reception of the electromagnetic pulse of the TDT. Figure 2 shows an analysis of the TDT waveform. The TDT waveform is represented by voltage as a function of time. The travel time, which is related to the soil volumetric water content, spans the beginning of the waveform to the voltage rise (Figure 2a), and it increases as the soil water content increases owing to the large dielectric constant of water. Further, the TDR also determines the travel time based on the waveform analysis. However, the waveform shape and the analysis method differ from those of TDT. These have been explained in detail by Blonquist et al. [35]. The new sensor outputs the digitalized travel time (*t*_D_). A travel time of zero corresponds to the maximum value of the digital number 4096. Therefore, *t*_D_ decreases as the travel time increases. The parameter σ_b_ is determined from the amplitude of the waveform after a voltage rise. Large σ_b_ materials cause propagating electromagnetic waves to attenuate, such that the amplitude of the waveform decreases as soil salinity increases. The voltage during the period after the rise in the waveform is digitalized to a value from 0 to 4096 (*V*_D_). 

The ceramic plate is in contact with the soil, which results in pore water exchange. When the system reaches equilibrium (no apparent exchange of pore water), the ψ of the soil and the ceramic plate are equal. If the relationship between the water content and the ψ of the ceramic plate is known, the soil ψ can be determined through water content measurements of the ceramic plate. Further, the measurement of the moisture content of the ceramic plate can be replaced by a capacitance measurement of the ceramic plate, which is strongly correlated with the moisture content. The capacitance of the ceramic plate is measured with the integrated circuit and converted to voltage. The frequency of the capacitance measurement is several dozens of MHz, and the capacitance range is within several dozens of pF. In addition, the voltage is digitized into numbers from 0 to 4096 (*C*_D_). The relationship between the *C*_D_ value and the soil ψ must be derived from calibration experiments. The thermistor outputs the voltage and is treated similarly. 

### 2.2. Sensor Calibration

Since the proposed sensor outputs digitalized values, it is necessary to obtain the relationship between these outputs and the target soil properties (the relationship between the temperature and thermistor output is provided by the producer). Therefore, we performed a series of calibration experiments. 

#### 2.2.1. Relationship between the Sensor Outputs and the Dielectric Constant/Matric Potential

TDR and TDT determine ε_b_ and convert it to θ using an empirically obtained relationship explained by Topp et al. [36]. Although this empirically obtained relationship can be applied to most soils, the relationship is soil-specific and does not work for some soils [37]. Therefore, it is more effective to convert the sensor output to ε_b_ instead of θ and use the conversion best suited for field soil.

The relationship between the sensor output *t*_D_ and ε_b_ and that between the sensor output *C*_D_ and ψ (kPa) were obtained simultaneously from a single experiment by comparing the sensor outputs with the reference sensors. The reference sensors were commercially available TDT sensors (SDI-12 soil moisture, Acclima, Meridian, ID, USA) and ψ sensors (ML-2600AES tensiometer, mol, Tokyo, Japan; MPS-6, Meter Group, Pullman, WA, USA). The proposed and reference sensors were placed in a plastic box with an inner length, width, and height of 30 cm, 20 cm, and 10 cm, respectively. Three sets of sensors and three plastic containers were used. The boxes were filled with three soils to confirm that the relationships were independent of the soil type. Toyoura sand, a soil collected from the Gifu University experimental field (hereafter, GU soil), and Andisol, whose textures were sand, sandy clay loam, and sandy loam, respectively. The soil texture, bulk density, and saturated hydraulic conductivity of each soil sample are listed in Table 1. GU soil contains a relatively large ratio of clay, whereas Andisol is a volcanic soil with a significant soil organic matter (SOM) content and a small bulk density. The soils were initially saturated and air-dried at a constant temperature of 20 °C. An electric fan was used to blow air across the soil surface to enhance the soil water evaporation. The control and data storage of the new sensor were conducted using a personal computer. A datalogger (CR1000, Campbell Scientific, Logan, UT, USA) was used for data collection from the SDI-12 soil moisture and tensiometer, and a datalogger (ZL6, Meter Group) was used for data collection from the MPS-6. The experiment was performed for 21 days, and each sensor measurement was performed every hour. The ε_b_ measured with the SDI-12 soil moisture and the ψ measured with the tensiometer and MPS-6 were compared with the sensor outputs *t*_D_ and *C*_D_ to establish the relationships.

#### 2.2.2. Relationship between Sensor Output and Electrical Conductivity

The calibration of the proposed sensor for σ_b_ was performed using measurements conducted in KCl solution. The sensor was placed in a plastic column with an inner diameter of 15 cm and a height of 18 cm. The column was filled with 0.0001 mol L^−1^, 0.00025 mol L^−1^, 0.005 mol L^−1^, 0.01 mol L^−1^, 0.05 mol L^−1^, and 0.1 mol L^−1^ concentrations of KCl solution, and new sensor measurements were performed in each solution. The electrical conductivity of each solution (mS m^−1^) was measured using a multiple water quality meter (MM-60R, DKK-TOA, Tokyo, Japan). The measurement results were then compared with the output value *V*_D_ of the new sensor. 

An additional experiment was conducted with KCl solution containing Toyoura sand to confirm that the calibration equation obtained from the above experiment with KCl solutions works for soil. The sensor was fixed in space within a plastic column (with an inner diameter of 8.3 cm and a height of 17 cm) filled with sand. The concentration of KCl solution was set at 0.1 mol L^−1^, and a known amount of KCl solution was added to the sand to achieve θ values of 0.15 m^3^ m^−3^, 0.20 m^3^ m^−3^, 0.25 m^3^ m^−3^, and 0.30 m^3^ m^−3^. The new sensor measurements were performed for each soil sample with a different θ value, and the sensor outputs were converted to σ_b_ using the calibration equation obtained from the above experiment (Equation (5)). Furthermore, the electrical conductivity of the KCl solution was measured using the MM-60R, and the σ_b_ of the sand (mS m^−1^) was calculated using the equation proposed by Hilhorst et al. [38]:

(1) $$\sigma_{b}=\frac{\sigma_{w}\left(\varepsilon_{b}-\varepsilon_{0}\right)}{\varepsilon_{w}}$$

where σ_w_ is the electrical conductivity of the KCl solution (mS m^−1^), ε_b_ is the dielectric constant of the soil, ε_0_ is the dielectric constant of the soil when its electrical conductivity is zero (which can be treated as that of oven-dry soil), and ε_w_ is the dielectric constant of water (≈80). The ε_b_ values were calculated backward from the θ value using Topp’s equation [36].

(2) $$\theta =4.3\times 10^{-6}\varepsilon_{b}{}^{3}-5.5\times 10^{-4}\varepsilon_{b}{}^{2}+2.92\times 10^{-2}\varepsilon_{b}-5.3\times 10^{-2}$$

The ε_0_ value was calculated using Equation (2) for θ = 0. Furthermore, we compared the σ_b_ measured using the proposed sensor with that calculated using Equation (1). 

### 2.3. Evaluation of Temperature Dependence of Sensor Outputs

The temperature dependence of soil sensors is often a problem owing the dynamics of soil temperature variation. The temperature dependence of the proposed and reference sensors was evaluated in a laboratory experiment. The new sensor, SDI-12 soil moisture, and MPS-6 were placed in plastic boxes with an inner length, width, and height of 30 cm, 20 cm, and 10 cm, respectively, and these were filled with Andisol with θ values of 0.20 m^3^ m^−3^, 0.40 m^3^ m^−3^, and 0.60 m^3^ m^−3^. Each plastic box was placed in a constant-temperature chamber, and the temperature was increased from 15 °C to 45 °C in 10 °C increments. We increased the chamber temperature when the sensor thermistor temperature reached the set chamber temperature. Each sensor was measured before changing the chamber temperature.

### 2.4. In Situ Sensor Evaluation

The in situ sensor performance evaluation was conducted in an experimental field at Gifu University (35°46′19.17″ N, 136°73′93.25″ E). The experimental field was maintained in bare soil. The proposed sensor was inserted at depths of 10 cm and 20 cm. The sensor board was placed perpendicular to the ground surface to prevent the obstruction of water and heat flow. The two sensors were connected to wireless communication modules (LoRa module), which sent the measured data to the master node located 40 m away from the field via LoRa communication. The master node then uploaded the data to the cloud via cell phone communication. A weather station (ATMOS 41, Meter Group) and a ZL6 datalogger were installed in the field to obtain weather data. New sensor and weather station data were collected every 15 min. The in situ evaluation began on 1 July 2021 and continued until 26 January 2022. A liquid nitrogen fertilizer was applied to the field on 11 November to determine whether the new sensor could capture an increase in σ_b_ caused by the liquid fertilizer.

## 3. Results and Discussion

### 3.1. Sensor Calibration

#### 3.1.1. Relationship between Sensor Output and Dielectric Constant

**Figure 3:**
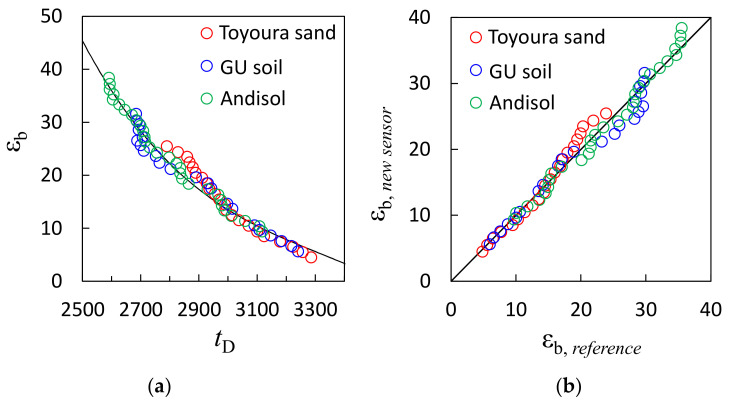
(**a**) Relationship between dielectric constant (ε_b_) measured with a reference sensor and *t*_D_ measured with the new sensor, and (**b**) comparison between ε_b_ measured with the reference sensor (ε_b*, reference*_) and the new sensor (ε_b*, new sensor*_). The different colored plots represent the different soil types, i.e., Toyoura sand, Gifu University experimental field soil (GU soil), and Andisol.

The relationship between the *t*_D_ of the new sensor and the ε_b_ measured with the SDI-12 soil moisture is shown in Figure 3a. The different colored plots represent the different soil types. The ε_b_ value of each soil showed ranges of 4–25, 6–32, and 9–38 for the Toyoura sand, GU soil, and Andisol, respectively. The associated *t*_D_ values were distributed between 2590 and 3290. Although a minor difference was observed in the ε_b_ ranges, the relationship between *t*_D_ and ε_b_ was consistent for all soils. Therefore, the relationship was independent of the soil type. We approximated this relationship using the following cubic equation:

(3) $$\varepsilon_{b}=-3.96\times 10^{-8}t_{D}{}^{3}+3.93\times 10^{-3}t_{D}{}^{2}-1.33t_{D}+1521.82$$

The coefficient of determination *R*^2^ for Equation (3) was 0.98. Figure 3b compares the ε_b_ values determined using the reference and proposed sensors. The two values were consistent and distributed around the 1:1 line. Slight errors were observed when the θ of each soil was relatively large because of the non-uniform distribution of water in the soil after the initial saturation process. Furthermore, the drying process may have homogenized the variation in the θ distribution, which resulted in better agreement at small θ values. The root-mean-square error (RMSE) and mean absolute percentage error (MAPE) were 1.74% and 6.7%, respectively. The output *t*_D_ of the proposed sensor can be converted to ε_b_ using Equation (3). The θ can be calculated from the ε_b_ by selecting an appropriate equation from the literature; for example, Topp et al.’s equation [36] can be used for most mineral soils and Miyamoto et al.’s equation [37] can be used for volcanic ash soils. Deriving a soil-specific equation to convert ε_b_ to θ will result in the best accuracy.

#### 3.1.2. Relationship between Sensor Output and Matric Potential

**Figure 4:**
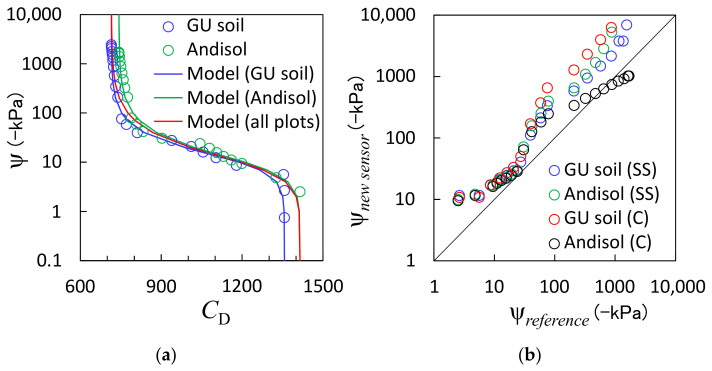
(**a**) Relationship between the soil matric potential (ψ) and the *C*_D_ measured using the reference and proposed sensors, respectively, and (**b**) comparison between the ψ measured with the reference sensor (ψ*_reference_*) and the proposed sensor (ψ*_new sensor_*). The different colored plots in panel (**a**) represent the Gifu University experimental field soil (GU soil) and the Andisol, and the solid lines indicate fitted models (Equation (4)) with the plots of either soil and with all plots. The different colored plots in panel (**b**) represent the GU soil and the Andisol with the sensor-specific (SS) or common (C) parameters of Equation (4).

Figure 4a shows the relationship between the *C*_D_ of the proposed sensor and the ψ measured using a tensiometer and an MPS-6. The tensiometer and MPS-6 had measurable ranges of ψ. We used the tensiometer-measured ψ larger than −100 kPa and MPS-6-measured ψ smaller than −100 kPa as the reference values. The *C*_D_ value of the proposed sensor and the reference sensor-measured ψ values did not respond to soil drying, that is, they were almost constant, in Toyoura sand. It is reportedly challenging to maintain a hydraulic connection to the pore water in course soil using the ψ sensors in porous media [26,28], and the same phenomenon occurred in the Toyoura sand. Therefore, Figure 4a only shows the results for the GU soil and Andisol. The *C*_D_ values were initially constant when the soil was wet (the ψ was greater than −5 kPa) before dynamically decreasing when the ψ reached −5 kPa as a result of drying. The *C*_D_ decreased continuously until the ψ reached −100 kPa. However, the *C*_D_ value decreased only slightly after the ψ reached −100 kPa. A minor difference was observed between the results from the two soils when the ψ was less than −100 kPa. Except for the previously mentioned loss of hydraulic connections in sand, ψ sensors are unlikely to exhibit soil dependence in porous media. Therefore, this difference may be because of individual sensor variability caused by minor differences in the ceramic plate structure and contact between the ceramic plate and the capacitance. Because the relationship between *C*_D_ and ψ is similar to the soil water retention curve, which represents the relationship between θ and ψ, we used the modified van Genuchten model [39], which is a commonly used model for soil water retention curves, to express these relationships:

(4) $$\psi =-\frac{1}{\alpha }{\left[{\left(\frac{C_{D}-C_{D,\;\min}}{C_{D,\max}-C_{D,\min}}\right)}^{\frac{n}{1-n}}-1\right]}^{1/n}$$

where *C*_D,max_ and *C*_D,min_ are the maximum and minimum values of *C*_D_, respectively, and α and *n* are curve shape factors. The four parameters obtained by the fitting are listed in Table 2. For the *C*_D,max_ and *C*_D,min_ values, we selected the maximum and minimum *C*_D_ values measured in the respective soils. The parameters of the two sensors in the GU soil and the Andisol were different in that the GU soil sensor showed lower values for the same potential below −100 kPa and above −10 kPa. Therefore, while it would be desirable to obtain sensor-specific parameters for each sensor by calibration, it requires considerable effort. Additionally, little variation was observed in the range of −100 kPa to −10 kPa, which is the dominant range in the actual field. Therefore, the common parameters for all the sensors were derived by fitting all the data plots. In this case, the minimum and maximum values measured in the two soils were selected for *C*_D,max_ and *C*_D,min_.

Figure 4b compares the ψ values determined with the new sensor and Equation (4) to the reference values. The two ψ values were consistent between −100 to −10 kPa, whereas the new sensor overestimated the ψ when the ψ was smaller than −100 kPa because the changes in the *C*_D_ values become smaller when the ψ < −100 kPa compared with those when the ψ > −100 kPa. However, the ψ range in the field is generally greater than −100 kPa. Therefore, we can conclude that the new sensor performs very well. The ψ values determined with the sensor-specific parameters and those determined with the common parameters were consistent when the ψ was above −100 kPa. While the errors with the common parameters increased when the GU soil was below −100 kPa, they decreased for the Andisol, which we considered to be coincidental. The ψ varies in order scale, so that the RMSEs of the logarithmic values of ψ were calculated. The RMSE was 0.57 for the GU soil with the soil-specific parameters, 0.60 for the Andisol with the soil-specific parameters, 0.88 for the GU soil with the common parameters, and 0.05 for the Andisol with the common parameters. The new sensors can measure the ψ between −10,000 kPa and −10 kPa, and the accuracy of the ψ measurements was particularly good between −100 kPa and −10 kPa.

In this study, we calibrated the new ψ sensor by comparing it with a commercialized ψ sensor (an MPS-6) during the soil air-drying process. Although this method is simple, it has certain disadvantages, such as the time-consuming nature of the soil air-dying process and the measurement errors of commercial sensors. Thus, sensor calibration with a pressure plate apparatus such as the one used by Noborio et al. [26] may yield better results.

#### 3.1.3. Relationship between Sensor Output and Electrical Conductivity

**Figure 5:**
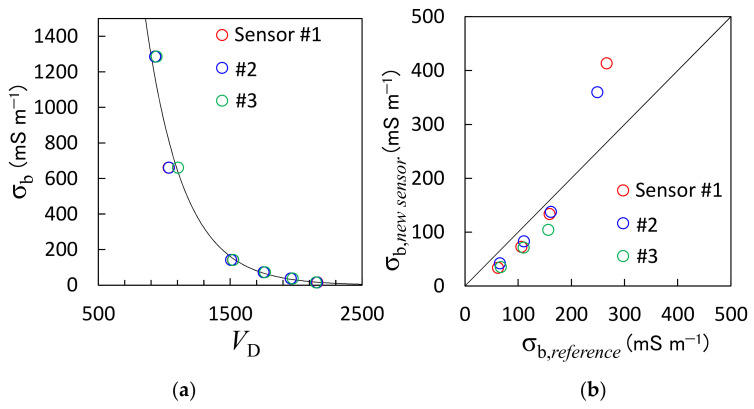
(**a**) Relationship between the electrical conductivity (σ_b_) of potassium chloride (KCl) solution measured with the reference sensor and the *V*_D_ measured with the proposed sensor, and (**b**) comparison between the σ_b_ of the Toyoura sand calculated with Equation (1) (σ_b,*reference*_) and the σ_b_ determined using the proposed sensor (σ_b,*new sensor*_). The different colored plots represent different sensor numbers.

The relationship between the *V*_D_ and σ_b_ for the KCl solutions is shown in Figure 5a. The σ_b_ values of the 0.1 mol L^−1^, 0.05 mol L^−1^, 0.01 mol L^−1^, 0.005 mol L^−1^, 0.00025 mol L^−1^, and 0.00010 mol L^−1^ KCl solutions measured with the multiple water quality meter were 1285 mS m^−1^, 661 mS m^−1^, 140 mS m^−1^, 72 mS m^−1^, 37 mS m^−1^, and 1 mS m^−1^, respectively. The *V*_D_ values decreased as the σ_b_ increased, and the relationship between the *V*_D_ and σ_b_ was expressed as an exponential function (denoted by the solid line in Figure 5a):

(5) $$\sigma_{b}=2.92\times 10^{4}\exp\left(-3.46\times 10^{-3}V_{D}\right)$$

The *R*^2^ for Equation (5) was 0.98. Notably, the ε_b_ values were also measured using the proposed sensor during the experiment, and it was found that the ε_b_ values were almost 80 (εb of water) for the 0.01 mol L^−1^, 0.005 mol L^−1^, 0.00025 mol L^−1^, and 0.00010 mol L^−1^ KCl solutions, but greater than 90 for the 0.1 mol L^−1^ and 0.05 mol L^−1^ KCl solutions. Furthermore, TDR sometimes fails to accurately determine the ε_b_ in large σ_b_ materials because the decrease in voltage amplitude causes ambiguous travel times (see Figure 2) [40]. The TDT system implemented in this study encountered a similar problem. 

Figure 5b compares the σ_b_ values of the Toyoura sand determined using Equation (1), which is assumed to be a reference, and those determined using the new sensor and Equation (5). The σ_b_ value increased from 61 mS m^−1^ to 266 mS m^−1^ as the θ increased. The reference and proposed sensor-determined σ_b_ values were consistent at σ_b_ values smaller than 200 mS m^−1^, whereas the larger σ_b_ values were slightly overestimated by the proposed sensor. However, the reference σ_b_ values that were determined using the Hilhorst model were not necessarily accurate since the Hilhorst model is less accurate at high salinity [38,41]. Therefore, it cannot be judged whether the sensor overestimated the value or whether the reference values were too small. The RMSE was 62 mS m^−1^. Although the large σ_b_ values were mismatched, the new sensor can determine the σ_b_ value of the soil with sufficient accuracy. 

### 3.2. Temperature Dependence

**Figure 6:**
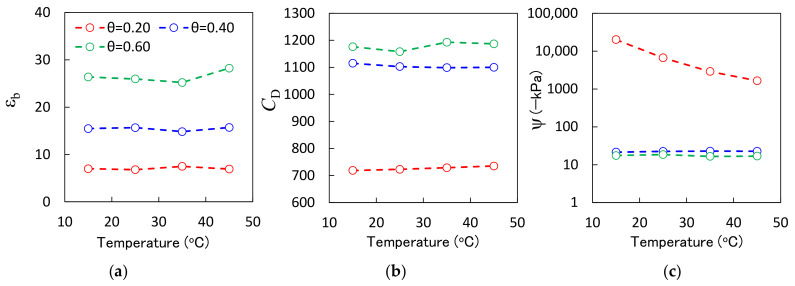
(**a**) The new sensor-measured dielectric constant (ε_b_), (**b**) digital values of the new sensor-measured capacitance (*C*_D_), and (**c**) the new sensor-measured matric potential (ψ) as functions of the temperature. The colored plot indicates the different water content (θ) of the Andisol, i.e., 0.20 m^3^ m^−3^, 0.40 m^3^ m^−3^, and 0.60 m^3^ m^−3^.

Figure 6 shows the ε_b_, *C*_D_, and ψ values of the proposed sensor as functions of the temperature. The ε_b_ values measured with the new sensor varied slightly with temperature. However, there was no clear trend (Figure 6a). For example, the ε_b_ value of θ = 0.60 m^3^ m^−3^ Andisol decreased from 26.3 to 25.2 as the temperature increased from 15 °C to 35 °C, but increased to 28.3 as the temperature increased from 35 °C to 45 °C. The dielectric constant of water decreased as the temperature increased [15]. In addition, Andisol is known to have large amounts of bound water, which is strongly adsorbed by the soil particles. Bound water therefore has a lower dielectric constant value compared with free water [42]. The ratio between this bound water and free water in the soil pores is partly controlled by the temperature [43]. Moreover, it is possible that water redistribution in the container may have influenced the measurements, thereby resulting in a complicated trend in the effects of the temperature on the ε_b_. Although the ε_b_ showed a complex temperature dependence, the ε_b_ fluctuation was at most 3.1, whereas the equivalent change in the θ calculated using Equation (2) was only 0.028 m^3^ m^−3^. Therefore, the temperature dependence of the ε_b_ need not be considered.

As the temperature increased, the *C*_D_ value of θ = 0.20 m^3^ m^−3^ Andisol increased from 719 to 735, and that of θ = 0.40 m^3^ m^−3^ Andisol decreased from 1115 to 1110. The ψ converted from *C*_D_ showed little change for θ = 0.40 m^3^ m^−3^ and θ = 0.60 m^3^ m^−3^ Andisol (Figure 6c). The fluctuation in the ψ in these soils was less than 2 kPa. However, the ψ value of θ = 0.20 m^3^ m^−3^ showed a dynamic increase from −20,133 kPa to −1649 kPa as the temperature increased, although the *C*_D_ value showed a slight increase because the minor temperature dependence of the *C*_D_ value is amplified by conversion using Equation (4) when the ψ is small, that is, when the soil water content is small. A similar phenomenon was reported for the MPS-6 [20], and it is a common weakness of capacitance and porous medium-based ψ sensors. Therefore, the temperature dependency of the ψ must be considered when a sensor is used in dry conditions. 

### 3.3. Field Evaluation

**Figure 7:**
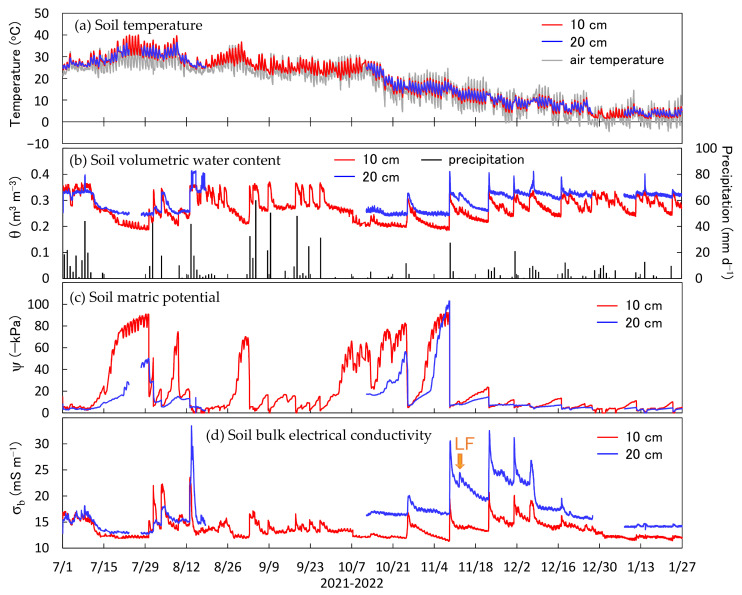
(**a**) Time series of soil properties measured with the new sensor in the Gifu University experimental field. The panels present (**a**) soil and air temperatures, (**b**) soil volumetric water content and precipitation, (**c**) soil matric potential, and (**d**) soil bulk electrical conductivity. The “LF” in panel (**d**) indicates liquid fertilizer application.

Figure 7 shows the in situ soil temperature, θ, ψ, and σ_b_ measured with the proposed sensor. The θ was calculated using Equations (2) and (3), and the ψ and σ_b_ were calculated using Equations (4) and (5), respectively. The parameters were determined from all the plots (Table 2). The sensors were installed at depths of 10 cm and 20 cm. The 20 cm depth sensor showed missing data from 18 August to 12 October 2021 and from 27 December 2021 to 7 January 2022 owing a malfunction of the wireless communication module. The 10 cm sensor, whose communication module functioned correctly, performed measurements throughout the period without any data loss. While the communication stability is an issue for future consideration, the sensor itself was stable and could perform measurements for a long period.

The soil temperature at depths of 10 cm and 20 cm showed fluctuations similar to those of the air temperature, which gradually decreased from approximately 40 °C to 5 °C from July to January. The temperature 10 cm from the soil surface had a larger amplitude compared with that at a 20 cm depth. The θ ranged between 0.18 m^3^ m^−3^ and 0.41 m^3^ m^−3^ throughout the experimental period, increasing with rainfall and decreasing with evaporation and infiltration. The value at 20 cm was approximately 0.05 m^3^ m^−3^ higher than that at 10 cm because a depth of only 10 cm was more likely to be affected by surface evaporation and rainfall drainage. The ψ varied from −2 to −103 kPa and increased to a value near zero immediately after rainfall before decreasing during subsequent drying. The ψ rarely fell below −100 kPa. As for the soil water content, the soil at a depth of 10 cm was more prone to drying compared with that at a depth of 20 cm, which indicated a lower matric potential. The σ_b_ ranged between 11 mS m^−1^ and 33 mS m^−1^, and the trend was similar to that of the soil water content. These results are satisfactory because the σ_b_ is controlled in part by the θ. The effect of liquid fertilizer applied on 11 November was difficult to determine because of the strong influence of the θ on the σ_b_. However, the decrease in the σ_b_ at a depth of 10 cm immediately after 11 November was milder than that during a similar drying process. Additionally, the σ_b_ at a 20 cm depth remained high from 22 November to 6 December, but a sudden decrease on 6 December was observed following rainfall owing to liquid fertilizer transport in the soil. As described above, the new sensor accurately captured phenomena in the field and, hence, can be considered to provide highly reliable data.

**Figure 8:**
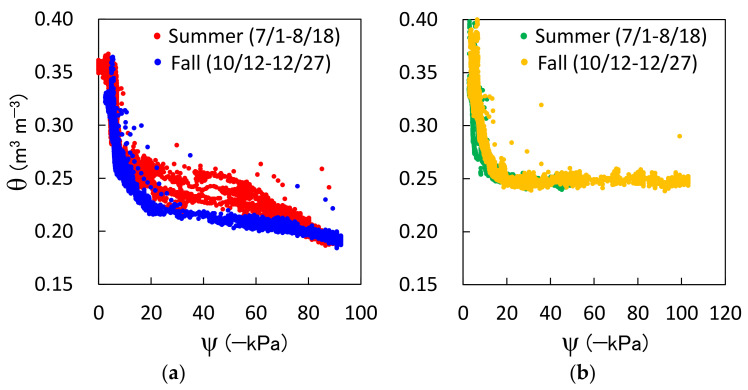
In situ water retention curves were obtained with the new sensors installed at depths of (**a**) 10 cm and (**b**) 20 cm. The different colored plots indicate the different periods: summer (from 1 July to 18 August 2021) and fall (from 12 October to 27 December 2021).

Because the new sensor can determine the θ and ψ in the field simultaneously, it can obtain the in situ soil water retention curve, shown in Figure 8. The curves for each depth were constructed for two different periods: summer (from 1 July to 18 August 2021) and fall (from 12 October to 27 December 2021). At a depth of 10 cm, the θ varied from 0.19 m^3^ m^−3^ to 0.37 m^3^ m^−3^ and the ψ varied from −70 kPa to −3 kPa with wetting and drying. At a depth of 20 cm, the θ varied from 0.24 m^3^ m^−3^ to 0.41 m^3^ m^−3^ and the ψ varied from −103 kPa to −3 kPa. The θ decreased slightly at a depth of 20 cm after reaching 0.25 m^3^ m^−3^, whereas the ψ kept decreasing relatively dynamically. Such a difference between the 10 cm and 20 cm depths can be attributed to the compaction and distance from the surface. These results show that ψ is a better indicator of soil dryness than θ. Furthermore, dynamic hysteresis was observed during the summer at a depth of 10 cm, but was rarely observed at a depth of 10 cm in the fall. However, hysteresis was not observed at a depth of 20 cm because of the rapid drying speed of the 10 cm soil layer in the summer, which is associated with high air temperature and intensive solar radiation. This rapid drying leaves more isolated water in the soil at the same ψ. The in situ soil water retention curves differ from those obtained in the laboratory [44] and provide essential information for estimating soil water movement in the field.

## 4. Conclusions

This study proposed a new TDT-based sensor that can simultaneously determine the soil water content (θ), matric potential (ψ), electrical conductivity (σ_b_), and temperature for application in smart farming. A series of laboratory and field tests were conducted to evaluate the performance of the proposed sensor. Laboratory tests revealed the relationships between the digital values of the sensor outputs and the soil properties, as well as the temperature dependence of the sensor outputs. The field test measured the transient soil conditions using the sensor for a relatively long period. The results indicated that the sensor provides satisfactory performance with acceptable accuracy, small temperature dependence, and a long lifetime. Additionally, the proposed sensor can provide vital information for the in situ soil water retention curve owing to its ability to simultaneously determine θ and ψ. We believe that the novel sensor proposed in this study can contribute to a wide range of studies on topics including smart farming, soil mass, and heat transfer.

## Figures and Tables

**Table 1:** Sand, silt, and clay percentages, soil organic matter (SOM) content, bulk density, and hydraulic conductivity of Toyoura sand, soil collected from the Gifu University experimental field (GU soil), and Andisol.

	% Sand	% Silt	% Clay	SOM Content (kg kg^−1^)	Bulk Density (kg m^−3^)	Hydraulic Conductivity (m s^−1^)
Toyoura sand	100.0	0.0	0.0	0.00	1340	1.2 × 10^−4^
GU soil	61.7	16.1	22.2	0.07	1080	1.5 × 10^−5^
Andisol	66.3	27.5	6.2	0.23	820	4.5 × 10^−6^

**Table 2:** Obtained parameters for the modified van Genuchten model that expresses the relationships between the capacitance digital value and the matric potential of the new sensor (Equation (4)). The parameters for the Gifu University experimental field soil (GU soil), the Andisol, and both soils (all plots) are shown.

	*C*_D,max_	*C*_D,min_	α (kPa^−1^)	*n*
GU soil	1357	715	0.086	2.32
Andisol	1415	743	0.100	2.11
All plots	1415	715	0.105	2.04

## Data Availability

Not applicable.
